# Mirtazapine Creating “Miracles” in Psychotic Depression With Catatonia

**DOI:** 10.7759/cureus.9863

**Published:** 2020-08-19

**Authors:** Rikinkumar S Patel, Nikhila Veluri, Geetika Verma

**Affiliations:** 1 Psychiatry, Griffin Memorial Hospital, Norman, USA; 2 General Medicine, American University of Integrative Sciences, St. Michael, BRB

**Keywords:** major depression, psychotic depression, mood disorders, catatonia, management, mirtazapine, antidepressants

## Abstract

Catatonia is commonly seen in patients with mood disorders and schizophrenia. The treatment of catatonia requires immediate attention as delayed care resulted in malignant catatonia. The first-line treatment for catatonia is benzodiazepines (BZDs) with rapid improvement. First-generation antipsychotics (FGAs) increase the risk of neuroleptic malignant syndrome and so are avoided in catatonic patients. Second-generation antipsychotics (SGAs) are recommended for treatment in catatonic patients. Treatment for catatonia due to depression includes serotonin reuptake inhibitors (SSRIs). When an individual manifests catatonia during an episode of depression with psychotic features, it is valid to administer both SSRIs and SGAs. Relatively very few studies have examined the use of atypical antidepressants, such as mirtazapine, and so we present a case of catatonia due to severe depression with psychotic features that improved significantly after the introduction of mirtazapine. Despite the beneficial effects of mirtazapine in psychotic depression and catatonia, it is underutilized due to the scarcity of literature. We recommend future clinical studies to evaluate mirtazapine’s "miracle" effects, particularly in such patients presenting with psychotic depression and catatonia.

## Introduction

Catatonia is seen in patients with schizophrenia and mood disorders, and less commonly in other psychiatric illnesses like post-traumatic stress disorder (PTSD), delirium, and dissociative disorders [1]. The prevalence of catatonia is about 10%, ranging from 7.6% to 38% in the acutely ill psychiatric inpatients, and about 20%-50% of these patients have depressive disorders, 28% showed mixed mood features of mania and depression, and 30% have schizophrenia [1-3]. A decrease in dopaminergic and gamma-aminobutyric acid (GABA) neurotransmission and impairment of N-methyl-D-aspartate (NMDA) signaling due to an excess of glutaminergic activity is the underlying mechanism to cause catatonia [4].

Heterogenicity of complex clinical symptoms and psychopathological conditions associated with catatonia often cause challenges in diagnosis and management, and so clinicians often call it a "catatonic dilemma" [5]. Few scales are available to assist clinicians in the neuropsychiatric examination, including the use of Kanner Scale and Bush-Francis Catatonia Rating (BFCR) Scale in conjunction with or without a benzodiazepine (BZD) testing method [2,6]. It is vital to manage catatonia optimally, as untreated catatonia is associated with significant morbidity and mortality due to the risk of neuroleptic malignant syndrome (NMS) and malignant catatonia [4]. Unresolved catatonia for four days imposes the risk of permanent disability [7].

BZDs are the first choice in catatonia management (efficacy of 60%-80%) as they act by decreasing NMDA receptor activity and simultaneously increasing GABA activity. Electroconvulsive treatment (ECT) is preferred in patients with high fever, delirium, greater physiological risk, or non-respondents to BZD, and has efficacy of 53%-100%. BZDs and ECT may be used concurrently but should be cautious; as past studies have found that BZD can increase the seizure threshold, whereas some recent studies found no significant impact on seizure threshold or duration [6]. When an individual manifests catatonia during an episode of depression, it is valid to administer selective serotonin reuptake inhibitors (SSRIs). Relatively very few studies have examined the usage of atypical antidepressants, such as mirtazapine, and so we present a case of catatonia due to severe depression with psychotic features, which improved significantly after the introduction of mirtazapine in existing psychotropic regimen.

## Case presentation

A 22-year-old African American male patient with no past psychiatric history was admitted to Griffin Memorial hospital (GMH) on emergency detention status for worsening depression with catatonic behavior and inability to care for self. During the initial psychiatric evaluation, the patient was catatonic and mute and responding to internal stimuli. The patient had poor posturing with waxy flexibility and poor eye contact. As the patient was brought by his mother, she provided the collateral information including the patient’s major recent stressor of losing his father due to alcohol intoxication in January 2020. The mother reported that the patient had made passive suicidal ideations, and endorsed visual hallucinations of images of his father, and appeared to be responding to internal stimuli. The patient endorsed paranoia and trespassed a property in a neighborhood that led him to the county jail for 1.5 months. Following his release from the jail, the patient had isolated himself from his family, and became unresponsive and mute. The patient did not take care of himself and had a very poor appetite with a significant 35-pound unintentional weight loss in the past three months.

The patient was born and raised in Oklahoma and was the oldest of two siblings. His childhood was uneventful, without any trauma. His relationship with his deceased father was "rocky". He graduated from high school and about to start working for "Amazon" before his arrest for trespassing. He has no significant past medical or surgical history. Besides his father’s alcohol use disorder, there is no other family history of psychiatric disorders. The patient has never received past inpatient/outpatient psychiatric care, and has no past suicide attempts, nor had suicidal and homicidal ideations. He does not have a history of substance or alcohol use.

On admission, the patient was started on fluoxetine 20 mg by mouth (PO), every morning (qAM) for depression, haloperidol 5 mg PO twice a day (BID) for psychosis, lorazepam 1 mg PO three times a day (TID) for catatonia, and benztropine 1 mg PO BID for prophylaxis of extrapyramidal symptoms (EPS). As per nursing report, the patient had eaten 20% of his meals following the administration of lorazepam. On day 3, the patient was mute, responded to internal stimuli, and refused to participate with the treatment team meeting. Haloperidol was increased to 10 mg PO BID.

Laboratory reports with complete blood count, comprehensive metabolic profile, lipid panel, hemoglobin A1c, and thyroid and renal function, and urine drug screen revealed no acute abnormalities. The next day, the patient continues to be non-verbal with waxy flexibility and posturing, and so lorazepam was increased to 1.5 mg PO TID. Subsequently, the patient was selectively mute with the treatment team and later responded to simple one-step commands. Per nursing reports, the patient had approached the pharmacy desk for his medications compared to his prior four days where the nurse had to go to the patient’s room for his medications. However, the patient continues to have a blunted affect, selective mutism, and psychomotor inactivity with waxy flexibility; therefore, lorazepam was increased to 2 mg PO TID, haloperidol was increased to 15 mg PO BID for psychosis, and fluoxetine was increased to 40 mg PO qAM for depression.

On day 5, nurses reported that the patient had been verbalizing in short answers and went to the basketball arena to play with other peers, and so the patient was tapered off lorazepam over the next five days, and simultaneously fluoxetine was titrated to 60 mg PO qAM. On day 11, the patient verbalized in short sentences with the treatment team for the first time and agreed with the plan of a long-acting injectable (LAI): haloperidol decanoate 150 mg intramuscular (IM) and then every four weeks. Mirtazapine 15 mg PO every nighttime (qHS) was added to augment antidepressant action and improve appetite. On day 15, the patient demonstrated significant improvement as he was able to maintain a good conversation with the treatment team and stated that his psychotropic regimen worked well for his depression rating it 3 to 4 out of 10 (was 10 out of 10 on admission). The patient also discussed his short-term goals of resuming playing music and start working to sustain his finances, and for the first time showed a willingness to speak with his mother and family. The patient had been eating 70% portions of his meals, participating in group and milieu therapy, and was taking care of his daily activities. Mirtazapine was titrated to 30 mg PO qHS and on day 19, the patient rated his depression 0 out of 10 and appreciated the treatment team for helping him with his depression and current situation. The patient had a linear and goal-directed thought process, with an improved insight and judgment, and agreed to be compliant with his psychotropic regimen by following up at psychiatric outpatient.

## Discussion

The underlying neurobiology for catatonia is GABA and dopamine hypoactivity, and glutamate hyperactivity [8]. The most widely accepted treatment for catatonia includes the use of BZDs with lorazepam as the most commonly used BZD as they have been positive GABA receptor modulators along with decreased expression, activity, and signaling of glutamate neural connections, thereby decreasing catatonic symptoms [9]. Antipsychotics are not recommended for the treatment of catatonia due to the risk of NMS; however, antipsychotics are administered to patients with an underlying history of psychosis or those who present with psychotic symptoms [8-10]. First-generation antipsychotics (FGAs) exhibit a higher potency on the dopamine d2 receptors, potentially increasing the risk for NMS and EPS, and so second-generation antipsychotics (SGAs) are preferred [2]. Pelzer et al. conducted a systematic review of catatonia treatment and found that clozapine outperformed other SGAs by demonstrating an 85% positive response [11]. In catatonia caused by clozapine withdrawal, clozapine is the first-line treatment. Other beneficial SGAs for catatonia include aripiprazole and olanzapine. Aripiprazole is a partial agonist at the dopamine d2 receptor, decreasing catatonia symptoms, without increasing the risk of EPS and NMS. Low-potency SGAs, particularly olanzapine, are preferred for catatonia since serious risks are associated with high-potency antipsychotics like risperidone [12].

Very few case reports have discussed the benefit of mirtazapine in psychotic depression. In a case report by De Mello, a 58-year-old female with severe psychotic depression showed a remission of delusional and depressive symptoms with the addition of mirtazapine 60 mg/day and haloperidol 10 mg/day, and was in remission after nine months of stabilization [13]. Furthermore, Ruberto et al. published three cases where the addition of mirtazapine in patients with psychotic depression alleviated their symptoms including delusions, and the patients remained asymptomatic for two years [14]. A case by Cheng and Shen involved a 42-year-old female without past psychiatric history, who was in shock after the sudden loss of her husband and presented with catatonic behavior to the hospital. Her catatonia improved with injectable diazepam 20 mg/day and olanzapine 10 mg/day over 10 days, but her depression and guilty delusion persisted. The addition of mirtazapine 60 mg/day improved her psychotic depression gradually [15].

In our case report, the patient had shown a profound improvement after tapering off lorazepam and administering haloperidol LAI, and most important is augmenting the antidepressant action of fluoxetine with mirtazapine. Mirtazapine is an atypical antidepressant with pronounced antihistaminic activity at low doses, and at higher doses it has more noradrenergic activity and antagonist effects on alpha-2-adrenergic receptors, histamine h1 receptors, and serotonin receptors (5-ht2a, 5-ht2c, 5-ht3). Antagonism of the 5-ht3 receptors reduces dopamine activity in the mesolimbic system, thereby reducing psychotic symptoms [14]. Inhibition of the 5-ht2a and 5-ht2c receptors increases the dopaminergic activity in some regions of the brain, thereby reducing catatonic symptoms [16]. Also, BZD in a psychotropic regimen improves both catatonia and depression, and should be continued for at least three to six months to prevent relapse/recurrence [17]. Few atypical antipsychotics/SGAs demonstrated efficacy in treating catatonic symptoms through similar mechanisms exhibited by mirtazapine (Table 1) [18].

**Table 1:** Mechanism of atypical antipsychotics in reducing catatonic symptoms ^a ^Antagonistic activity at dopamine d2 and/or serotonin 5-ht2a: activates the mesocortical dopamine pathway, specifically the nigrostriatal and mesolimbic pathway that may contribute to decreasing catatonic symptoms; and antagonistic activity at 5-ht2c: increase in dopamine in the prefrontal cortex that likely contributes in reducing catatonic symptoms. ^b^ Agonistic activity at dopamine d2 and/or serotonin 5-ht1a: similar to d2 and 5-ht2a antagonistic activity. They contribute to activating the mesocortical dopamine pathway, specifically the nigrostriatal and mesolimbic pathway that leads to the reduction of catatonic symptoms.

	Antagonistic activity^a^			Agonistic activity^b^	
	Dopamine d2	5-ht2a	5-ht2c	Dopamine d2	5-ht1a
Aripiprazole				x	x
Quetiapine	x	x			x
Olanzapine	x	x			
Risperidone	x	x			
Ziprasidone	x	x			x
Clozapine		x	x		
Amisulpride	x				
Asenapine	x	x			x
Lurasidone	x	x			x
Iloperidone		x			
Cariprazine		x		x	x

## Conclusions

BZD is the treatment of choice for treating catatonia, and SGAs should be added in patients presenting with psychotic symptoms. SGAs like olanzapine and aripiprazole should be preferred first due to less dopamine antagonism and to avoid the worsening of catatonic symptoms. Among antidepressants, mirtazapine should be preferred as it causes “miracle” by significantly improving psychotic depression and catatonia. Lack of studies and case reports on the efficacy of mirtazapine has led to underutilization of this medication in such cases.
